# Diagnostic Utility of the Impact of Event Scale–Revised in Two Samples of Survivors of War

**DOI:** 10.1371/journal.pone.0083916

**Published:** 2013-12-31

**Authors:** Nexhmedin Morina, Thomas Ehring, Stefan Priebe

**Affiliations:** 1 Department of Clinical Psychology, University of Amsterdam, Amsterdam, The Netherlands; 2 Institute of Psychology, University of Muenster, Muenster, Germany; 3 Unit for Social and Community Psychiatry, Barts’ and the London School of Medicine and Dentistry, Queen Mary University of London, United Kingdom; University of Pennsylvania, United States of America

## Abstract

The study aimed at examining the diagnostic utility of the Impact of Event Scale-Revised (IES-R) as a screening tool for post-traumatic stress disorder (PTSD) in survivors of war. The IES-R was completed by two independent samples that had survived the war in the Balkans: a sample of randomly selected people who had stayed in the area of former conflict (n = 3,313) and a sample of refugees to Western European countries (n = 854). PTSD was diagnosed using the MINI International Neuropsychiatric Interview. Prevalence of PTSD was 20.1% in the Balkan sample and 33.1% in the refugee sample. Results revealed that when considering a minimum value of specificity of 0.80, the optimally sensitive cut-off score for screening for PTSD in the Balkan sample was 34. In both the Balkan sample and the refugee sample, this cut-off score provided good values on sensitivity (0.86 and 0.89, respectively) and overall efficiency (0.81 and 0.79, respectively). Further, the kappa coefficients for sensitivity for the cut-off of 34 were 0.80 in both samples. Findings of this study support the clinical utility of the IES-R as a screening tool for PTSD in large-scale research studies and intervention studies if structured diagnostic interviews are regarded as too labor-intensive and too costly.

## Introduction

Post-traumatic stress disorder (PTSD) is a common and disabling disorder with onset after traumatic experiences [1]. According to the fourth edition of the Diagnostic and Statistical Manual of Mental Disorders (DSM-IV) [2], diagnostic criteria for PTSD require the onset of 17 characteristic symptoms following exposure to an extreme stressor (Criterion A1) and a reaction to that stressor that involves fear, helplessness, or horror (Criterion A2). Further, post-traumatic symptoms must be present for more than one month and include intrusive recollections of the traumatic event (Criterion B; at least 1 symptom), avoidant symptoms (Criterion C; at least 3 symptoms), and hyperarousal symptoms (Criterion D; at least 2 symptoms) [2]. The very recently published fifth edition of the DSM (i.e., DSM-5) [3] proposes four distinct diagnostic clusters instead of three: re-experiencing symptoms (at least 1 symptom), avoidant symptoms (at least 1 symptom), negative alterations in cognitions and mood (at least 2 symptoms), and arousal symptoms (at least 2 symptoms). Additionally, DSM-IV Criterion A2 (i.e., and a reaction to that event that involves fear, helplessness, or horror) has been deleted, due to its low utility in predicting the development of PTSD. In both editions of the DSM, symptoms of PTSD must persist for more than one month and must cause clinically significant distress or impairment in functioning.

Different structured diagnostic interviews can be used to diagnose PTSD. However, all of them require a trained interviewer and are time consuming for both the interviewer and the interviewee. For large-scale research studies and for assessing changes in PTSD during the course of treatment, such interviews can be too labor-intensive and costly. Briefer self-report methods would be preferable. A commonly used scale to assess PTSD symptoms in clinical and non-clinical settings is the Impact of Event Scale- Revised (IES-R). The original impact of Event Scale (IES) consists of seven items measuring intrusions and eight items measuring avoidance related to a negative event [4]. Weiss and Marmar [5] revised the questionnaire to better match diagnostic criteria for PTSD as specified in the DSM-IV [2]. Accordingly, in addition to intrusion and avoidance items, the IES-R includes items capturing hyperarousal as the third main symptom cluster of PTSD according to the DSM-IV. The IES-R consists of 22 items and participants are asked to rate each symptom as to how distressing it has been during the past seven days. Weiss and Marmar [5] further modified the response format of the IES-R from a 4-point (0, 1, 3, and 5) to a 5-point (0, 1, 2, 3, and 4) response format. Yet, the IES-R includes one item not listed in the DSM-IV (“I felt as if it hadn’t happened or wasn’t real”) and does not assess three symptoms listed in the DSM-IV PTSD. The IES-R has demonstrated good psychometric properties [6] and is currently one of the most widely used measures to assess posttraumatic stress symptoms [7]. Although the questionnaire was originally not intended to be used for screening and/or the assessment of a diagnosis of PTSD [6], its good psychometric properties and its wide availability make it a promising brief self-reported measure for assessing PTSD.

Several studies to date have examined the utility of the IES-R to identify individuals with PTSD. Creamer, Bell, and Failla [8] used the IES-R in a community sample of 159 male Vietnam veterans with varying degree of PTSD symptomatology. A cut-off of 1.5 (equivalent to a total score of 33) was found to show the best agreement with PTSD diagnosis established by another self-report measure, the PTSD checklist (PCL) [9] (sensitivity = 0.91, specificity = 0.80). Asukai et al. [10] reported a cut-off of 30 for the Japanese version of the IES-R against structured clinical interviews in a sample of 73 survivors of arsenic poisoning and a second sample of 86 earthquake survivors (sensitivity = 0.83 and 0.75, specificity = 0.85 and 0.72, for the first and second sample respectively). Rash and colleagues [11] reported a cut-off score of 22 as the best agreement with the PTSD diagnosis as assessed with the Clinical-Administered PTSD Scale (CAPS) [12] among 124 traumatized substance dependent individuals (sensitivity = 0.92, specificity = 0.57). Finally, Adkins et al. [13] examined the diagnostic utility of the IES-R among 239 trauma-exposed American undergraduate students. A cut-off score of 44 was found to show best agreement with PTSD diagnosis as assessed with the CAPS (sensitivity = 0.67, specificity = 0.94). Sveen and colleagues [14] evaluated the Swedish version of the IES-R to screen for PTSD, yet the Swedish version of the IES-R uses a different response format (0, 1, 3, 5) than the English version (0, 1, 2, 3, 4) and therefore its diagnostic utility cannot be compared to the above mentioned studies.

While the IES-R holds promise as a screening instrument, prior research has used rather small convenience samples that ranged from 60 to 239 (and a combined total sample size of 595), which does not appear appropriate to establish reliable cut-off scores. The established cut-off scores differed considerably, ranging from 22 to 44. In addition, one of the studies [8] used a cut-off on a second questionnaire measure, the PCL, to establish PTSD caseness, rather than structured clinical interviews. Furthermore, in order to be used in screening of a diagnosis among different populations, the measure in question should be robust across diverse samples [15]. The only study providing a cross-validation in a second independent sample showed that the high values for sensitivity and specificity from the first sample could not be replicated in the second one [10].

In summary, evidence on the utility of the IES-R as a screening instrument for PTSD is still inconclusive. In the current study, the usefulness of the IES-R for assessing PTSD was investigated in two independent large samples who had survived the war in Ex-Yugoslavia with the aim of replicating findings of the first sample in the second one. Given that the above mentioned studies on the diagnostic utility of the IES-R have provided different cut-off scores that ranged from 22 to 44, we conducted our study without an a priori hypothesis.

## Methods

### Procedure

The data were obtained in a multi-center study conducted in 2005 and 2006 that assessed long-term mental health outcomes in people who had experienced potentially traumatic events during the war in Ex-Yugoslavia and had either stayed in the countries of conflict (Bosnia-Herzegovina, Croatia, Kosovo, Macedonia, and Serbia) or taken refuge in Western European countries (Germany, Italy, and the United Kingdom (UK)). Details about the rationale of the study and its methods have been described in detail elsewhere [16]–[18]. Participants were included if they had been born within the territory of the former Yugoslavia; were between 18 and 65 years old; had experienced at least one war-related potentially traumatic event; had experienced the last war-related event at or after 16 years of age; had no severe learning difficulty and no mental impairment due to a brain injury or other organic cause. In the countries of former Yugoslavia, participants were recruited using a multi-stage probabilistic sampling frame and random walk approach in administrative regions that had been directly exposed to war activities. First, 20% of administrative regions in each Balkan country were randomly chosen among those directly exposed to war. Then, three localities with a minimum population of 3,000 each were randomly selected in these administrative regions in each country. Finally, streets in these localities were randomly identified. In a particular street, every fourth household was selected until a maximum of 15 interviews for that street was reached.

In Germany, Italy, and the UK the sampling procedure was less rigorous and had to be adapted for various reasons. Most importantly, in these countries there were no areas with a sufficient density of survivors of war in Ex-Yugoslavia to use a random walk method for recruitment. In Germany and Italy potential interviewees were identified through local resident registers and snowball sampling. Potential participants on resident registers were sent invitation letters. In the case of no response, participants were sent two additional reminder letters. In the absence of accessible resident registers in the UK, potential interviewees were contacted through community organizations and snowball sampling.

The total refusal rate in the countries of former Yugoslavia was 29.9%. In the countries of Western Europe, the rates of individuals who participated in the study was much lower (52.9%), and we cannot establish the response rates for snowball sampling.

### Participants

A total of 3,313 participants in the countries of Ex-Yugoslavia and 854 refugees in Western European countries were interviewed. Due to missing data, 20 participants from the Balkan sample and 58 participants from the refugee sample were excluded from the analyses. Accordingly, the analyses involving the IES-R were conducted with 3293 participants from the Balkan countries and 796 refugees. In the Balkan sample 53.8% of participants were female as compared to 51% in the refugee sample. The mean age of participants was 42.5 (SD = 12.0) in the Balkan sample and 41.6 (SD = 10.8) in the refugee sample. Other socio-demographic and trauma related characteristics are reported in Table 1. Participants in both samples reported exposure to at least one war-related traumatic event that they experienced at age 16 years or older and that can be regarded as equivalent to the stressor criterion 1A of PTSD described by DSM-IV.

**Table 1:** Socio-demographic, Trauma-related, and IES-R-related Characteristics Among both Samples.

	Balkan countries (*N* = 3313)	Western countries (N = 854)
**Socio-demographic characteristics**		
**Gender**		
Female	1793 (53.8)	438 (51.3)
Male	1529 (46.2)	416 (48.7)
**Age**	42.5 (12.0)	41.6 (10.8)
**Marital status**		
Married/cohabiting	2328 (70.3)	652 (76.3)
Single	606 (18.3)	89 (10.4)
Divorced/separated	176 (5.3)	76 (8.9)
Widowed	202 (6.1)	37 (4.3)
**Education level attained**		
None or primary education	1007 (30.4)	188 (22.0)
Secondary school	1618 (48.8)	354 (41.5)
Vocational/tertiary	688 (20.8)	312 (36.5)
**Employment status**		
Employed	1188 (35.9)	351 (41.1)
Unemployed	1545 (46.6)	438 (51.3)
Retired	439 (13.3)	31 (3.6)
Training/education	141 (4.3)	34 (4.0)
**Trauma-related characteristics**		
Combat involvement	578 (17.4)	192 (22.5)
Number of pre-war traumatic events	0.7 (1.1)	1.1 (1.3)
Number of war traumatic events	4.2 (2.8)	6.8 (3.6)
Number of post-war traumatic events	0.6 (0.8)	1.1 (1.3)
Time since most traumatic war event (years)	8.1 (3.3)	10.5 (3.1)
**IES-R-related characteristics**		
IES-R total	24.2 (23.2)	31.8 (26.8)
IES-R-Intrusion	9.1 (9.0)	12.5 (10.5)
IES-R-Avoidance	8.8 (8.4)	11.2 (9.4)
IES-R-Hyperarousal	6.3 (6.9)	8.6 (8.2)

Note. Socio-demographic data (apart from age) and combat involvement are presented as *N* (*%*); age, trauma related characteristics (apart from combat involvement), and IES-R scores are presented as *M* (*SD*); *IES-R* = Impact of Event Scale-Revised.

### Ethics Statement

Written informed consent was obtained from participants before the interview. The study was approved by the Royal Free Medical School Research Ethics Committee (REC reference number 04/QO501/118).

### Measures

The *Life Stressor Checklist–Revised* [19] was used in an amended form to assess 24 potential types of war related traumatic events. Cumulative scores were calculated for pre-war, war and post-war experiences.

*The Impact of Event Scale – Revised* (IES-R) [5] was used among both war survivors on the Balkans as well as refugees to assess post-traumatic stress reactions. The responses of the 22 items range from 0 (“not at all”) to 4 (“extremely”). A detailed description of the IES-R was offered above. The authors reported high internal consistencies of the three subscales, with alpha coefficients ranging from 0.79 to 0.92, and high test–retest reliabilities, with correlation coefficients ranging from 0.51 to 0.92. The IES-R has been translated and validated for prior research in the countries of former Yugoslavia [20], [21]. In the current study, all participants identified at least one war-related event. After that, they were asked to rate each IES-R item with respect to the war-related traumatic event they described as most bothering. In this study, the IES-R had a high and similar internal consistency of the total scale as well as of the three subscales ranging from α = 0.92 to α = 0.95.

*The MINI International Neuropsychiatric Interview* (MINI) [22] was used to assess PTSD. The MINI is a structured diagnostic interview based on DSM-IV and ICD-10 criteria, which require exposure to an extreme event (Criterion A1), and a reaction to that event that involves fear, helplessness, or horror (Criterion A2), 13 symptoms on re-experiencing, avoidance, and hyperarousal, a minimum duration of symptoms of one month, and clear evidence of impairment in social or daily functioning. The MINI has demonstrated good reliability and validity in comparison with the Structured Clinical Interview for DSM-III-R (SCID) and Composite International Diagnostic Interview. Compared to the SCID, the module of PTSD demonstrated high inter-rater reliability (*k = *0.95), good test–retest reliability (*k = *0.73) and good values on sensitivity (0.85) and specificity (0.96) [22].

### Data Analysis

The prevalence rate of PTSD was calculated as the percentage of participants meeting criteria for this disorder according to the MINI at the time of survey. To analyze differences in traumatic experiences and the IES-R between groups, χ^2^ tests and t-tests were used depending on the type of data and using an alpha level of 0.05. Receiver Operator Curves (ROC) analyses were conducted in order to examine the extent to which the IES-R can accurately estimate diagnosis of PTSD. ROC analyses were conducted with the IES-R total score with regard to: *Sensitivity, Specificity, Overall Efficiency, Positive Likelihood Ratio (LR+), Negative Likelihood Ratio (LR-), and Area Under the Curve (AUC)* [*23*]. Following recommendations by Kraemer [15], [24], test statistics are presented as quality indices (i.e., kappa coefficients). These indices help better interpret and can adjust for optimal sensitivity (e.g, for screening purposes), specificity (e.g., differential diagnosis), and efficiency (overall agreement). The quality indices used are *κ* (1, 0) regarding sensitivity; *κ* (0, 0) regarding specificity; and *κ* (0.5, 0) regarding overall efficiency [15]. Kappa coefficients of 0.55 or higher can be interpreted as showing acceptable agreement [25]. In accordance with the emphasis of our study on screening, we aimed for an optimal value of sensitivity alongside a minimum value of specificity of 0.80. Results were analyzed using SPSS (version 18.0) and DAG_Stat software [24]. Primary analyses on the utility of the IES-R in identifying PTSD were conducted with participants from the Balkan countries. Findings were then cross-validated in the independent sample of refugees.

## Results

All participants reported exposure to at least one war-related potentially traumatic event (i.e., equivalent to the stressor criterion 1A of PTSD described by the DSM-IV). Table 1 presents trauma-related characteristics of both samples. The average time since the most traumatic event was 8.1 years (*SD* = 3.3) among participants in the Balkan countries and 10.5 years (*SD* = 3.1) among refugees. On average, refugees in the Western countries reported a significantly higher number of war-related potentially traumatic events (*M* = 6.76, *SD* = 3.62) than participants living in the Balkan countries (*M* = 4.17, *SD* = 2.79), *t*(4165) = 22.61, *p*<.001; d = 0.80). The most often reported war-related potentially traumatic events among both refugees and participants in the Balkan countries were “shelling or bombardment” (85.1% of refugees vs. 84.6% of participants in the Balkans), “lack of shelter” (64.5% of refugees vs. 51.4% of participants in the Balkans), “siege” (59.5% of refugees vs. 40.1% of participants in the Balkans), and “murder or death of a close person due to violence” (60.8% of refugees vs. 35.9% of participants in the Balkans). Additionally, participants in the Balkan countries reported on average 0.7 (*SD* = 1.1) and 0.6 (*SD* = 0.8) pre-war and post-war potentially traumatic events, respectively. The average pre-war and post-war potentially traumatic events among refugees was 1.1 (*SD* = 1.3) and 1.1 (*SD* = 1.3), respectively.

The prevalence rates for PTSD were 20.1% in the Balkan sample and 33.1% in the refugees (*χ* ^2^ = 65.8, *df* = 1, *p*<0.001). The values of internal consistency of the IES-R were high resulting in a value of *α* = 0.97 in both groups. Table 1 presents the distribution of the scores of the IES-R total and the three subscales among the groups. As compared to participants living in the Balkan countries, refugees reported significantly higher scores of the IES-R total as well as the subscales (all *ps.* <0.001; d = 0.30). Participants with PTSD reported significantly higher scores of the IES-R total (both *ps* <.001; both d = 1.92 for the Balkan sample and refugees; see Table 2).

**Table 2:** IES-R Scores Among Participants in the Balkan Countries and Refugees with and without PTSD.

Balkan group (*N* = 3311)	PTSD positive (*N* = 665)	PTSD negative (*N* = 2646)	*t*-test	*p*-value
IES-R total	52.2 (17.8)	17.2 (18.7)	43.39	<0.001
IES-R- Intrusion	19.8 (7.1)	6.4 (7.3)	42.32	<0.001
IES-R-Avoidance	17.8 (6.8)	6.5 (7.2)	36.23	<0.001
IES-R-Hyperarousal	14.7 (5.8)	4.2 (5.4)	43.58	<0.001
**Refugee group (** ***N*** ** = 854)**	**PTSD positive (N = 283)**	**PTSD negative (N = 571)**	**t-test**	**p-value**
IES-R total	57.9 (18.0)	20.1 (21.2)	24.39	<0.001
IES-R- Intrusion	22.3 (7.4)	7.9 (8.3)	24.00	<0.001
IES-R-Avoidance	18.9 (7.0)	7.6 (8.0)	19.57	<0.001
IES-R-Hyperarousal	16.4 (5.8)	4.8 (6.2)	25.85	<0.001

*Note. IES-R* = Impact of Event Scale-Revised; IES-R scores are presented as *M* (*SD*).

**Figure 1:**
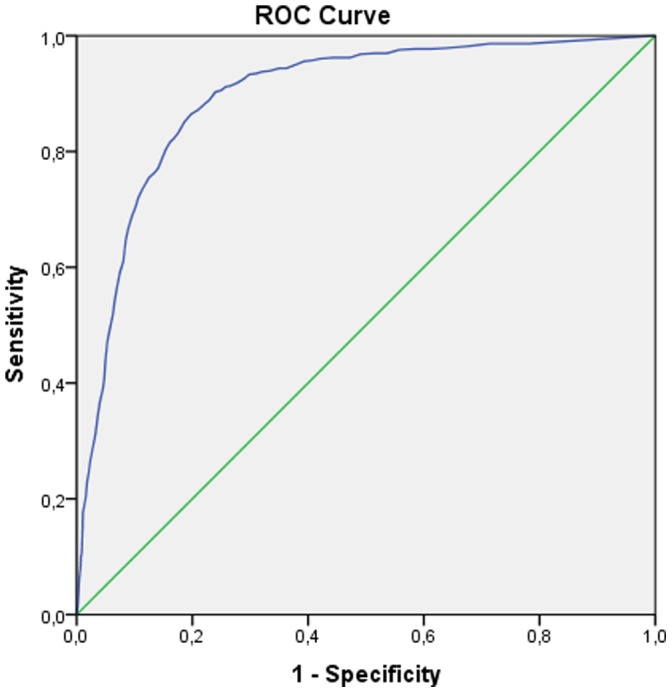
Receiver Operator Curves (ROC) showing the optimal IES-R scores for identifying diagnosable PTSD in the Balkan group.

**Figure 2:**
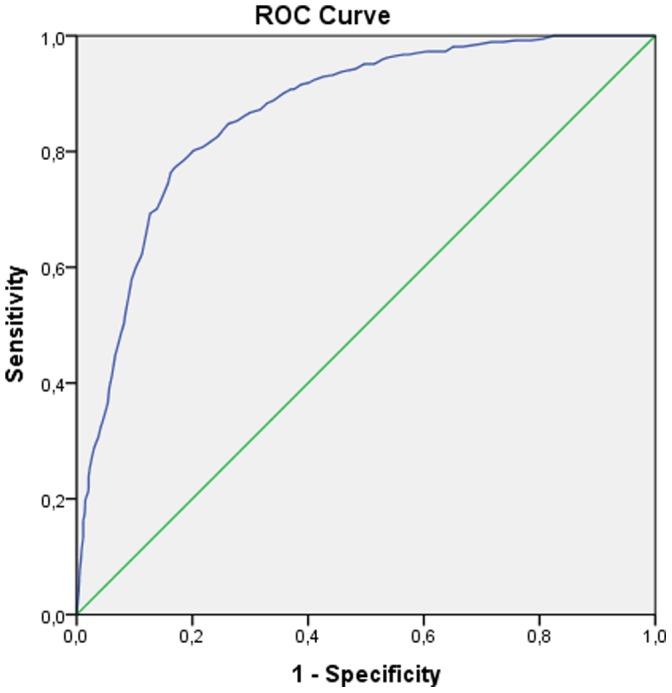
Receiver Operator Curves (ROC) showing the optimal IES-R scores for identifying diagnosable PTSD in the refugee group.

The receiver operator curves (ROC) for the Balkan and refugee samples are presented in Fig. 1 and Fig. 2, respectively. The area under the curve (AUC) that measures overall accuracy was 0.90 (95% CI: 0.88–0.91) and 0.87 (95% CI: 0.85–0.89) for the Balkan sample and the refugee sample, respectively. Table 3 presents the values of the ROC analyses. Cut-off scores from prior research (i.e., 22, 30, 33, and 44, respectively) are associated with good to excellent sensitivity (ranging between 0.74 and 0.94 for the Balkan countries and 0.81 and 0.95 for the refugee sample) as well as kappa coefficients for sensitivity (*κ* (1.0) ranging between 0.65 and 0.88 for the Balkan countries and 0.67 and 0.91 for the refugee sample). However, these cut-off scores were associated with rather low kappa coefficients for specificity *κ* (0,0), ranging from 0.27 to 0.52 for the Balkan sample and 0.31 to 0.54 for the refugee sample. Finally, in the Balkan sample, the kappa coefficient for overall efficiency *κ* (0.5,0) was satisfactory for the cut-off score of 44 only (0.58). In the refugee sample, the kappa coefficient for overall efficiency *κ* (0.5,0) was satisfactory for the cut-off scores of 44 and 33 (0.56 and 0.60, respectively).

**Table 3:** Cut-off Scores and Discriminative Ability of the IES-R.

	Cut-off	*SE*	*κ* (1.0)	*SP*	*κ* (0,0)	Efficiency	*κ*(0.5, 0)	*AUC*
**Balkan group**								
IES-R total	49	0.65	0.56	0.92	0.57	0.86	0.56	0.78
	48	0.67	0.58	0.91	0.56	0.86	0.57	0.79
	47	0.69	0.60	0.90	0.55	0.86	0.58	0.80
	46	0.70	0.61	0.90	0.54	0.86	0.58	0.80
	45	0.72	0.64	0.89	0.53	0.86	0.58	0.81
	44	0.74	0.65	0.89	0.52	0.86	0.58	0.81
	43	0.76	0.67	0.87	0.50	0.85	0.57	0.81
	42	0.76	0.68	0.87	0.49	0.85	0.57	0.81
	41	0.77	0.67	0.86	0.47	0.84	0.56	0.82
	40	0.79	0.71	0.85	0.46	0.84	0.56	0.82
	39	0.80	0.73	0.85	0.46	0.84	0.56	0.82
	38	0.82	0.74	0.84	0.45	0.83	0.56	0.82
	37	0.82	0.74	0.83	0.43	0.83	0.55	0.83
	36	0.83	0.76	0.82	0.43	0.83	0.55	0.83
	35	0.85	0.78	0.81	0.42	0.82	0.54	0.83
	34	0.86	0.80	0.80	0.40	0.81	0.54	0.83
	33	0.87	0.81	0.79	0.39	0.81	0.52	0.83
	32	0.88	0.82	0.78	0.37	0.80	0.51	0.83
	31	0.89	0.83	0.77	0.37	0.79	0.51	0.83
	30	0.90	0.85	0.76	0.36	0.79	0.50	0.83
	22	0.94	0.88	0.68	0.27	0.73	0.44	0.81
**Refugee group**								
	49	0.75	0.63	0.86	0.59	0.83	0.61	0.81
	48	0.78	0.67	0.86	0.59	0.84	0.63	0.82
	47	0.79	0.67	0.86	0.59	0.84	0.63	0.82
	46	0.79	0.68	0.84	0.56	0.83	0.61	0.82
	45	0.80	0.68	0.84	0.55	0.83	0.61	0.82
IES-R total	44	0.81	0.69	0.82	0.54	0.82	0.60	0.82
	43	0.81	0.70	0.82	0.53	0.82	0.60	0.82
	42	0.83	0.72	0.81	0.52	0.82	0.60	0.82
	41	0.84	0.73	0.80	0.50	0.81	0.59	0.82
	40	0.84	0.73	0.80	0.49	0.81	0.59	0.82
	39	0.85	0.75	0.79	0.49	0.81	0.59	0.82
	38	0.86	0.76	0.77	0.46	0.80	0.57	0.82
	37	0.87	0.77	0.76	0.45	0.80	0.57	0.82
	36	0.88	0.78	0.76	0.45	0.80	0.57	0.82
	35	0.88	0.78	0.74	0.43	0.79	0.56	0.81
	34	0.89	0.80	0.74	0.43	0.79	0.56	0.82
	33	0.90	0.81	0.73	0.43	0.79	0.56	0.82
	32	0.91	0.82	0.72	0.42	0.78	0.55	0.82
	31	0.92	0.84	0.71	0.40	0.78	0.54	0.81
	30	0.92	0.84	0.71	0.40	0.77	0.54	0.81
	22	0.95	0.91	0.61	0.31	0.72	0.46	0.78

*Note. SE* = sensitivity; *SP* = specificity; *AUC* = Area Under the Curve; *κ* (1,0) = quality index of sensitivity; *κ* (0,0) = quality index of specificity; *κ* (0.5,0) = quality index of efficiency (Kraemer, 1992; Mackinnon, 2000).

Considering a minimum value of specificity of 0.80, the cut-off score of 34 provided the most optimally sensitive cut-off score for screening purposes in the sample of participants in the Balkan countries. In this sample, the cut-off score of 34 provided good values on sensitivity (0.86), specificity (0.80), and overall efficiency (0.81). The kappa coefficient for sensitivity was 0.80.

Among refugees, the cut-off score of 34 was also associated with good sensitivity (0.89), specificity (0.74), and overall efficiency (0.79). Similarly to the Balkan sample, the kappa coefficient for sensitivity was good (0.80). The lowest cut-off score in this sample to meet the above specified criterion of a minimum value of specificity of 0.80 was 40. This cut-off score had good values of sensitivity (0.84), specificity (0.80), and overall efficiency (0.81). The kappa coefficient for sensitivity was 0.73.

## Discussion

The findings of this study in two independent samples suggest that the IES-R can be effectively used as a screening instrument for PTSD. A cut-off score of 34 showed excellent sensitivity (0.89 and 0.86) as well as kappa coefficients used as quality indices sensitivity in both samples, whereas specificity was somewhat lower in the refugees than in the Balkan sample (0.74 vs. 0.80).

The optimally sensitive cut-off score identified in our study differs from the cut-off scores reported in prior research, yet it lies within the range of these cut-off scores (i.e., between 22 and 44). Our study extends prior findings by (1) comparing the IES-R with PTSD assessed via a structured clinical interview, (2) using much larger samples, (3) cross-validating the findings in two independent samples, and (4) using kappa coefficients as quality indices for sensitivity, specificity, and overall efficiency as suggested by Kraemer [15]. The use of kappa coefficients as quality indices enables adjustment for optimal sensitivity based on the aim of the application of the test in question. Corresponding to the aim of testing the efficiency of the IES-R as a screening tool, we theoretically aimed for an optimal value of sensitivity alongside a minimum value of specificity of 0.80. An additional value of kappa coefficients lies in the improved interpretation of levels of an acceptable agreement. The discrepancy between our results and prior findings on the utility of the IES-R [8], [10], [11], [13] may be a result of several factors. First, three of the four studies mentioned in the introduction [8], [10], [11] did not use kappa coefficients as quality indices for their calculation of the optimally efficient cut-off score. Adkins et al. [13] used the quality of efficiency *κ* (0.5, 0) as the key index of diagnostic utility and reported a cut-off score of 44 as optimally efficient. In fact, this cut-off score was the only score from prior research to be associated with satisfactory quality of efficiency *κ* (0.5, 0) in both our samples. It should be noted, however, that the aim of the study by Adkins et al. [13] was on overall efficiency that is rather associated with confirming a diagnosis and which is different to that emphasis in the current study that was on screening.

There is a lack of universal criteria available to establish the optimally sensitive or efficient cut-off score for assessing a diagnosis on a self-reported scale as the relative importance of sensitivity and specificity depends on the specific purpose of the assessment and the likely prevalence of the diagnosis [26], [27]. For screening purposes, high sensitivity is usually the most important way of assuring that as many individuals in need of treatment as possible are correctly identified [15]. Acceptable levels of specificity are then important to save limited resources for further assessment and treatment. For evaluating changes during the course of treatment, specificity may be relatively more important. To accommodate these different potential purposes of using the IES-R for assessing a PTSD diagnosis, the optimally sensitive or efficient cut-off score must be adjusted based on the purpose of the project (see Table 3). Our kappa coefficients regarding sensitivity and specificity indicate that the IES-R is a good screening tool for PTSD in the sense that it identifies the absolute majority of individuals with PTSD (i.e., 86% and 89% of them in the Balkan sample and refugee sample, respectively, see sensitivity values on Table 3) as well as the absolute majority of individuals without PTSD (i.e., 80% and 74% of them in the Balkan sample and refugee sample, respectively, see specificity values on Table 3). However, our study suggests that a cut-off score of 34 is less valid when it comes to differential diagnosis. In this regard, about 46% of individuals with an IES-R cut-off score of 34 did not meet criteria for PTSD according to the MINI. On the other hand, only about 5% of participants below an IES-R cut-off score of 34 met criteria for PTSD according to the MINI. Accordingly, if the aim of a given project is mainly the identification of individuals with PTSD, our results indicate that the cut-off score of 34 might be used reliably in achieving this goal. If the aim is, however, to weigh false positives and negatives equally, the optimally efficient cut-off score must be adjusted (see Table 3). Finally, the extent to which the IES-R is adequately sensitive to treatment change remains unknown and needs to be investigated in future research.

Another explanation for the discrepancy of our findings with previous results might be the notion that the optimally sensitive cut-off score may depend on the type of traumatization and other characteristics of the sample studied, such as time elapsed since the trauma [24]. Finally, the inconsistency might also be a result of the small sample sizes used in the previous studies.

The diagnostic utility of the IES-R as a screening tool for PTSD identified in this study is comparable with previous publications on screening instruments for PTSD among different populations. For example, Brewin [27] reported in his review of 22 instruments for post-traumatic reactions an average of sensitivity of 0.83 and specificity of 0.85. In sum, our results suggest that the IES-R is a useful instrument to screen for PTSD in war survivors.

The current study has a number of strengths. Results were based on a consistent methodology across several countries, including civilians and people with combat experience. The multi-stage probabilistic sampling frame and random walk approach applied in Balkan countries make it likely that the findings are representative for large populations in war-affected areas. All interviewers were well-trained researchers with a relevant professional background, were familiar with the given local context, and spoke the mother tongue of the interviewees. Most importantly, in contrast to earlier studies the diagnostic properties of the IES-R were established in two independent samples, and even the smaller of the two samples was significantly larger than the previously available samples on the association of IES-R scores and PTSD diagnosis combined. However, two main limitations are worth noting. Firstly, whilst the cut-off of 34 showed comparable values across the two samples, the lowest cut-off score to meet the previously specified criteria for this study in the refugee sample was higher than in the Balkan sample (40 vs. 34). Secondly, all participants had experienced war-related events in Ex-Yugoslavia and the results might not be generalizable to samples with other types of traumatic events. These limitations indicate that the optimally efficient cut-off score of the IES-R might vary depending on the characteristics of the specific population in question. It should be further noted that in resource-constrained countries screening can be beneficial only if its results can translate into actual treatment for those who screen positive [28], [29].

As mentioned above, DSM-IV PTSD criteria have been modified in the current version of the DSM (i.e., DSM-5). The first modification regarding the question what constitutes a traumatic event has no impact on the use of the IES-R items to screen for PTSD. However, the inclusion of three new symptoms in the DSM-5 (1. blame of self or others; 2. negative emotional state; and 3. reckless or destructive behavior) might influence the capability of the IES-R to screen for DSM-5 PTSD diagnosis. Future research needs to examine the extent to which the IES-R can be applied as a reliable screening instrument for PTSD according to the DSM-5 criteria.

When structured clinical interviews are not feasible or absorb inappropriate resources, IES-R scores can be used with reasonable accuracy to identify people with PTSD. This can apply to screening in populations and assessments in large-scale research studies. In both clinical and research settings, the IES-R has been reported to be one of the most frequently used measures [7], and results of the current study show that a cut-off score of 34 can be used to translate the IES-R scores into an assessment of a PTSD diagnosis. Examples could include surveys among populations that have been collectively exposed to potentially traumatic events such as armed conflicts, terrorist attacks, large-scale accidents, and natural disasters. One may conclude that existing IES-R scores can be used to screen for PTSD and underline the usefulness of the scale for research and clinical purposes.
